# Developing an online knowledge sharing platform and community of practice for health professionals: Experiences from C‐WorKS developed in North East England and Yorkshire during COVID‐19

**DOI:** 10.1111/hir.12519

**Published:** 2024-02-01

**Authors:** Peter van der Graaf, Andrea Burrows, Helen Park, Sarah Sowden

**Affiliations:** ^1^ Faculty of Health and Life Sciences, Department of Nursing Midwifery and Health Northumbria University Newcastle‐upon‐Tyne UK; ^2^ School of Health and Life Sciences, Department of Allied Health Professionals Teesside University Middlesbrough UK; ^3^ Public Health England North East and Yorkshire Newcastle‐upon‐Tyne UK; ^4^ Office of Health Inequalities and Disparities (OHID)/ Population Health Sciences Institute Newcastle University Newcastle‐upon‐Tyne UK

**Keywords:** health services research, internet, knowledge management, knowledge transfer, pandemic, public health

## Abstract

**Background:**

Although knowledge sharing online has been recognised as an important strategy for health professionals to apply research findings to their practice, limited research exists on how to develop and implement these platforms to help facilitate collaboration and knowledge sharing.

**Objectives:**

This study evaluated an online knowledge sharing platform and community of practice developed in the North East of England and Yorkshire during COVID‐19 to support UK health and care professionals to reduce the impact of the wider consequences of COVID‐19.

**Methods:**

Semi‐structured interviews with stakeholders (*n* = 8) and users of C‐WorKS (*n* = 13), followed by an online survey (*n* = 19) among a wider group of users to analyse knowledge use.

**Results:**

Interview and survey findings highlighted several strengths, weaknesses, opportunities and threats to support future development of online knowledge sharing platforms.

**Discussion:**

Online knowledge sharing supports six ‘pillars’ of successful research and innovation partnerships. This requires distributed forms of leadership and linking of different knowledge sharing strategies, and careful combination of platforms with communities of practice.

**Conclusion:**

Online knowledge sharing provides pragmatic and timely strategies for health professionals in the UK to apply research evidence to their practice. Our study provides generalisable, practical insights in how to develop and implement a knowledge sharing platform.

**Key Messages**

- Online knowledge sharing platforms provide health professionals with a pragmatic and timely strategy for applying research evidence and other types of knowledge to their practice.
- Successful online knowledge sharing platforms should be built on six pillars: purpose, leadership, inclusion and personalisation, skills and capability, data and technology infrastructure, and evidence‐based decision‐making.
- As platforms develop, distributed leadership among member organisations is needed to increase capacity and motivation for sustained involvement, and to reduce organisational resistance to practice recommendations (new 7th pillar).
- Combining data platforms with communities of practice reduces information overload, increases accessibility and traffic, while supporting members in applying knowledge and collaborating across organisational boundaries.

## BACKGROUND

Literature on knowledge management is traditionally concerned with knowledge creation and dissemination within organisations (Varun Grover, 2001). However, with the widespread distribution of high‐speed Internet, the role of online social spaces in knowledge management has grown rapidly (Cheung et al., 2013). Online communities provide useful platforms for knowledge extraction, exchange, and creation, both within and across organisational boundaries (Baker‐Eveleth et al., 2005). They enable members from a range of organisations and background to contribute, discuss, and share their knowledge with others. This results in knowledge becoming more collaborative and integrated (Chen & Hung, 2010). Online communities mitigate the hinderers of distance, time limitations due to busy life, and members isolation (Jesionkowska, 2020; Pesare et al., 2017).

Much of this research originated from studies in computer science and technology, with very limited applications found in health, in spite of evidence that online knowledge translation technique can foster interactions between various health professionals and assist in the sharing of ideas and knowledge within the health field (Mairs et al., 2013). Their systematic review of the literature indicated the potential of a diverse range of online strategies, such as wikis, discussion forums, blogs, and social media to data/knowledge management tools, virtual communities of practice, and conferencing technology. These strategies provide pragmatic and timely means to selectively transfer valuable research to policy makers, healthcare providers, the public and other stakeholders in order to plug existing gaps in knowledge (Ho et al., 2003) and overcome geographical constrains.

However, online knowledge translation techniques also present challenges, for example, in access to and user knowledge of online technologies. In addition, information overload has been reported for clinicians when searching for health‐related information online (Hall & Walton, 2004). The proliferation of online knowledge require both time and proficiency from user to sift through the available information and apply this knowledge to their context (Eysenbach & Köhler, 2002). Therefore, there is a growing need for online knowledge exchange strategies that can share current, reliable, easily accessible information related to specific health domains.

Hardly any practical examples exist on how to develop and implement these strategies within a health context and often originate from outside the UK. For example, the SUPPORT (Support for People and Patient‐Oriented Research and Trials) Unit in Alberta developed an online Knowledge Translation Platform to assist local health researchers and systems with improving the quality and quantity of patient‐oriented research in Canada (Thomson et al., 2019). The unit developed a programme theory for the platform, consisting of capacity building activities, a Community of Practice, consultation services, and identifying contributions to science. These activities were found to help the local health system implement evidence with measurable positive health outcomes. However, community members remained hesitant about their capacity as individuals to design and perform important knowledge exchange activities independently. In addition, recommendations or practice changes were sometimes met with resistance at organisational or governmental levels (Kothari et al., 2015).

More recent research is available from the literature on online adult learning in Online Communities of Practice (OCOP) with various professional sectors increasingly using online communities to address their learning needs. For example, studies have looked at the characteristics of online communities and what may facilitate or hinder adults' engagement in these communities (Abedini et al. 2021). This research highlights the need to engage different levels of professional expertise and experience among members of these communities to encourage learning and motivate members to contribute. Therefore, more research is needed on how to develop and implement these platforms in practice within a UK health context to help knowledge and information professionals to facilitate online knowledge sharing. This paper addresses this gap by reporting and reflecting on the findings of an evaluation of an online knowledge sharing platform and community developed in the North East of England and Yorkshire (NE&Y).

### Introducing C‐WorKS

**FIGURE 1:**
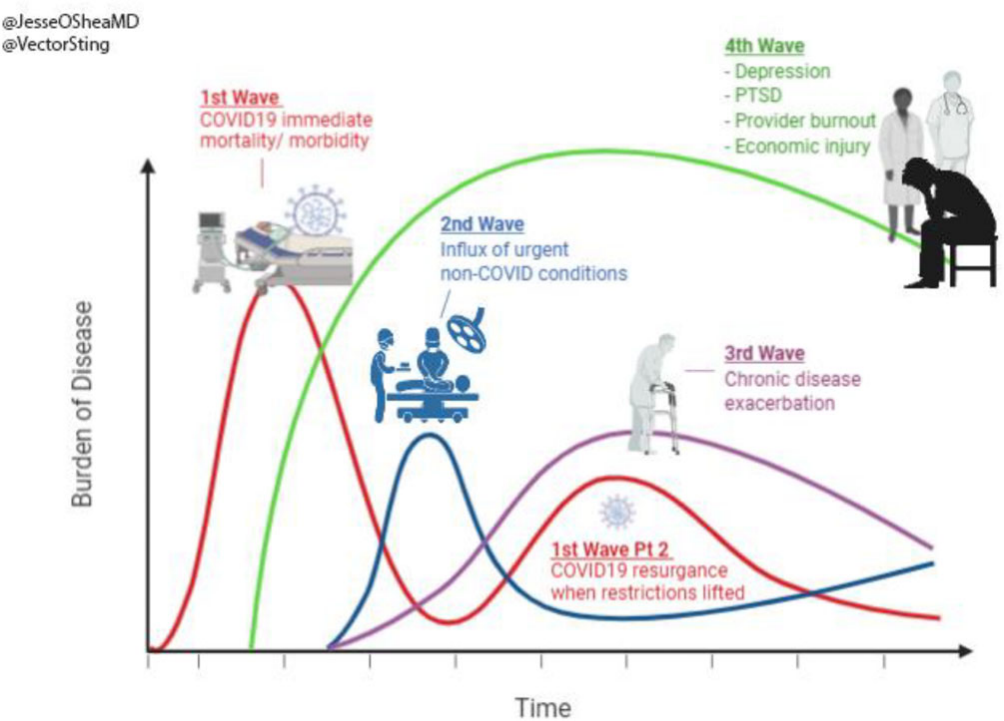
Waves of COVID‐19 related to burden of decease (credit @Vector Sing). [Colour figure can be viewed at wileyonlinelibrary.com]

The online knowledge sharing platform C‐WorKS was developed in early 2020 by a partnership of organisations including Public Health England (PHE), the National Health Service, academics and other public and third sector organisations in response to the COVID‐19 pandemic. Although, there was a considerable national response in the UK to managing high numbers of cases, complications, and deaths as a direct result of COVID‐19, the partnership expected that the COVID‐19 pandemic would also have far reaching impacts on non‐COVID related death and disease, as outlined in Figure 1.

The hub was developed in response to an urgent need identified across various health organisations in the NE&Y to collate and share new knowledge and intelligence created in response to COVID‐19 in the region, and to collaborate across organisational and sector boundaries. The lack of a sharing platform hindered learning at system level and the development of more effective, efficient and equitable responses to the pandemic. The primary aim of C‐WorKS was to build a regional repository of information and knowledge exchange and to encourage collaboration. This aim is reflected in the name; the acronym C‐WorKS stands for Covid‐consequences: Want it? OR Know it. Share it! It was envisioned that C‐WorKS would reduce duplication, highlight gaps, and promote information exchange.

The C‐WorKS platform hosts a plethora of information on non‐COVID impacts in a structured framework. The information is shared by users and on an ongoing basis; users can upload or describe information of work, and work in progress, share contact information, communicate with colleagues via a comments section and view shared information. Resources within the repository include data, analysis, research, and evidence, grouped by themes. An extensive scoping review carried out by Public Health England was used to determine the themes (reference withheld). In addition, C‐WorKS produced a regular email newsletter, regular KITS (Knowledge Intelligence Themed Summaries) shared via email, and a programme of regular events along the themes on the platform. ‘Soft’ work undertaken by the C‐WorKS team included making connections between relevant individuals from different organisations.

**FIGURE 2:**
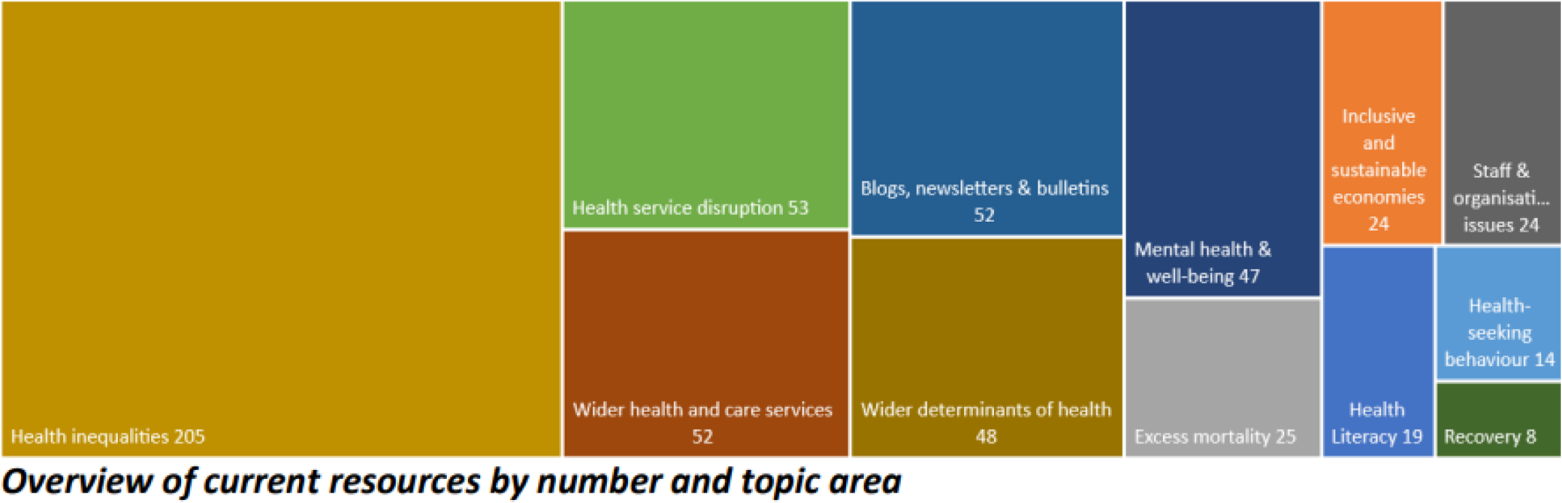
Overview of C‐WorKS resources by number and topic area (January 2023). [Colour figure can be viewed at wileyonlinelibrary.com]

The knowledge hub was launched on 1st June 2020, utilising PHE's existing KHub data platform, as a readily available and familiar resource for regional partners. Since the launch, C‐WorKS has been actively promoted at all‐staff events by senior staff across the region. Since the launch, 931 members have signed up to C‐WorKS, 357 members have visited in the past 6 months (132 in the past month), uploading 553 resources in several topic areas, as illustrated in Figure 2 (data till January 2023).

The C‐WorKS multi‐agency strategic Review and Act Group (RAG) was formed as part of the emerging management and governance structures for C‐WorKS. This group worked alongside the initial Management and Project Steering Group (MAPS). The roles, membership and remit of these groups developed over time. In part due to the academic representation on this group, the RAG recognised an opportunity to learn from the development and implementation of the platform and the difference the platform has made to members in sharing knowledge and intelligence and collaboration across the region. Anecdotal evidence suggested significant ‘outside platform’ activity, however, available monitoring data from C‐WorKS was not sufficient to capture this and other impacts (e.g., use of knowledge through C‐WorKS) and learning between stakeholders. Therefore, the group applied successfully to the NIHR Applied Research Collaboration (ARC) for the North East and North Cumbria (NENC) in 2020 for funding to conduct collaborative research between C‐WorKS partners and ARC NENC researchers, hosted by the Knowledge Mobilisation and Implementation Science theme of the NENC ARC.

## OBJECTIVES

The evaluation study aimed to:

- Examine the process of developing and embedding C‐WorKS to identify learning for practice partners and academics, to assess what worked and didn't work in developing and implementing the knowledge hub;
- Understand the impact of C‐WorKS on knowledge sharing outside the hub and how this sharing informed and changed practices across different organisations;
- Describe whether and how the concept of C‐WorKS could be adapted for other scenarios where enhanced sharing of information across a diverse network could be valuable.

## METHODS

The study applied a mixed methods design focusing on both the development and implementation process and outcomes of C‐WorKS, combining interviews with stakeholders, users, and a social network survey. Semi‐structured online interviews were undertaken with a sample of key stakeholders (*n* = 8) between October and December 2021 about their experiences of developing and implementing C‐WorKS, including key lessons, barriers and facilitators. These interviews were followed‐up by semi‐structured online interviews and email exchanges with a stratified sample of C‐WorKS users (January–February 2022; *n* = 13) to explore knowledge use and impact upon practice. The results of the interviews informed an online survey (*n* = 19) to conduct a social network analysis and map the reach and connectivity of the C‐WorKS community.

### Stakeholder and user interviews

Participants for the stakeholder interviews were purposively sampled for maximum variation, such as different settings and organisation types (Local Authorities, NHS Knowledge and Library Services, Office for Health Inequalities and Disparities (OHID; formerly Public Health England) and Academia) and levels of engagement with C‐WorKS. Participants included Knowledge and Intelligence Leads, Intelligence Analysts, Data Managers, Public Health Consultants, Clinical Librarians, and an Insight Coordinator from the Voluntary and Community Sector. Interviews took place on MS Teams and were audio‐recorded for transcription using STREAM. A topic guide was developed during the project's early scoping stages, informed by local knowledge focusing on early implementation, ongoing roll‐out, decision‐making processes, barriers and challenges, and any perceived outcomes (for organisations, staff and/or end users; see Appendices 1 and 2).

Participants for the user interviews were purposefully sampled from all registered C‐WorKS/ KHub users (*n* = 732) based on organisation type, region, job title and whether they submitted any enquiries or resources to the platform (level of engagement). This resulted in an initial sample of 30 users, who were invited by C‐WorKS leads by email for interviews. Four users consented to interviews and nine users provided written responses to the topic guide by email. The limited response to interview invites illustrates the significant pressures that local authority and NHS staff currently operate under, severely limiting participants' ability to engage in the research.

### Social network survey

To get a more detailed understanding of the impact of C‐WorKS on knowledge mobilisation outside the knowledge sharing hub, we employed a verified online survey to measure the extent of network ties between C‐WorKS users, in terms of reciprocal relationships and exchange of knowledge. The survey (see Appendix 3) is modelled on a stakeholder network survey developed by Dr Ruth Hunter from Queen's University Belfast and was applied in this research with her permission (Cheetham et al., 2019; StakeholderNet). The survey was administered in JISC online survey, inviting all registered users with C‐WorKS to participate by email. The survey was completed by 19. Given the low number of responses, the findings from the survey only provide an indication of sharing of knowledge outside the platform and the description of the network has been limited, including only qualitive descriptors.

### Data analysis

Interview transcripts were inputted in NVivo 12 for thematic framework analysis using an inductive approach based on Ritchie and Spencer (1994). This involves a series of processes to develop key themes from the data including familiarisation, indexing, framework development, mapping and interpretation. The survey data was inputted and analysed in SPSS28 using descriptive analysis and graphs. Data from the interviews and survey were triangulated through thematic framework analysis (Srivastava & Thomson, 2009) and informed by joint interpretation meetings with C‐WorKS partners, including co‐production of reports, and this paper.

## RESULTS

The findings from the interviews and survey indicate that C‐WorKS has emerged as a useful platform in the NE&Y for enabling the sharing of knowledge and intelligence about indirect impacts of COVID‐19. This includes both unseen positive impacts and concerns regarding access to and provision of key services, including the potential disproportionate impacts on non‐COVID illness and access to services for some groups, leading to increased inequalities.

Data analysis generated several main themes and highlighted key concerns. These can be briefly summarised as different strengths, weaknesses, opportunities and threats (SWOT). We will discuss each element of our SWOT analysis in turn below.

### Strengths

C‐WorKS was developed in record time, following a strategic scoping review. Strong network links across the region, the availability of an existing platform already actively used by one partner organisation (Public Health England), with buy‐in from senior members from the start, combined with pledged operational capacity for development and implementation, meant that C‐WorKS could be developed and implemented in 3 months. This rapid response undoubtedly contributed to the success of C‐WorKS, providing tailored data sources and a sharing platform for health professionals in need of urgent information due the unprecedented COVID‐19 pandemic when no such platform existed.

COVID has brought health and social care much closer together. It's brought clinicians and professionals together with finance and executives and lay members in ways that we haven't before. I think it's been about simultaneously expanding the networks and then utilizing those networks to push things like outputs from C‐WorKS into that space. (user)

The active governance structure which evolved during the early months of the venture, with close collaboration between the strategic Review and Action Group (RAG) and the operational Management And Project Steering group (MAPS) ensured shared decision making and team work to facilitate the rapid development, promotion and implementation of C‐WorKS, which is all the more remarkable given the in‐kind contribution of time from members of both groups. Instead of imposing a hierarchical structure with performance management, both groups worked in tandem, supporting each other's decision making.

You didn't have to have documents approved by the steering group [RAG] to be put on C‐WorKS, for example. It was much more seeking advice group, you know, a sense of direction. Have we got it? Are we advertising it to the right people? You know all those sorts of things. I don't know that there were any particularly hard decisions taken by the [RAG] group. (stakeholder)

With memberships of RAG and MAPS involving staff across different organisations in the NE&Y, the platform was able to work smoothly across organisational and sectorial boundaries, including NHS, PHE, Local authorities, research and academic partners, and the voluntary and community sector.

We've got some really good people on the group who are involved in other networks. That we would not, I mean, I would never come across some of the networks, so that works quite well. (stakeholder)

And having seen other people's expertise [in C‐WorKS] and that has probably led me to, you know, connect with people outside of the Review and Act as well. I think that's it's not often you get a forum where you can actually all collectively put your intelligence juices on the problem. We don't always have the answer, but it's interesting just to get those different perspectives. (stakeholder)

This new way of working manifested itself in two ways: 1) members developing their networks and engaging with other professionals they never worked with before; and, consequently, 2) making more use of existing data and intelligence from other sectors when making decisions.

It frustrates me as a leader in the NHS that my fellow NHS colleagues are less embracing of knowledge and intelligence that's out there; people tend to believe what's in front of them and don't tend to go looking for data [..], which means we're half blind: the interventions that we're designing are based on substandard insight. [..] these resources are so valuable, so keep going and know that people like me find them really valuable and do use them to try and push [them] out to really broad networks across health and care. (user)

### Weaknesses

At the same time, the goodwill and in‐kind contributions of RAG and MAPS members for the development and implementation of C‐WorKS poses a challenge for the sustainability of the platform. Lack of funding and work‐loaded time means that future development and operation of C‐WorKS is dependent on the generosity of staff time, which is unlikely to be limitless.

There are other parts of C‐WorKS, so the webinars—they've been brilliant but they are a resource by someone in my team who spends her time trying to make that happen. If I lose that member of staff, where does it go? I'm putting in the resource out of goodwill; it's not a funded position, no one pays for C‐WorKS; it's a collaboration. So, if I had to pull my team, I'm not sure who would take it on; I think that's a big problem. (stakeholder)

The current hosting platform for C‐WorKS is also not sufficiently user friendly, despite many improvements over time. While being a readily available platform for Office for Health Improvement and Disparities (OHID, formerly Public Health England) networks, the accessibility and navigation of KHub is perceived as limiting by C‐WorKS users, hampering them in sharing and promoting resources from C‐WorKS with their colleagues.

The wiki pages, I think they could have been better, but it was what was available to us at the time, and I think that might put users off: too many links to get pushing things out, getting registered and then navigating to pages. It's not particularly slick, but then, in terms of kind of early days of the pandemic, we didn't know how long the project was going to last for, so why build a whole new platform when you got something available with quite a multidisciplinary network already registered on the site. Made sense really, you know in a kind of pragmatic way. (stakeholders)

The limitations of the existing hosting platform and lack a funded staff capacity and resources to develop C‐WorKS, has impacted on user engagement with C‐WorKS. For example, some users admitted to not bothering with logging in on the platform and, instead, waiting for the weekly newsletter summaries to arrive in their email boxes to access and share relevant information with colleagues.

The only time I tend to access it is when I get on the Public Health Intelligence North East (PHINE) network; you get emails and there's the odd updates that come through, I tend to use those. When we get a summary, sometimes there are C‐WorKS links on there. That's when I tend to use it: I just have a quick scan to see whether or not, I think there's something that's useful for my network. I'll tend to click in it, have a read of it. And then if I think it is useful, I'll then push it out through the Clinical Commissioning Group (CCG) team briefs, that kind of thing. I might ask a member of my team to pull it into a word document or if it's something that I can save as a PDF, I'll get it circulated that way. (user)

**FIGURE 3:**
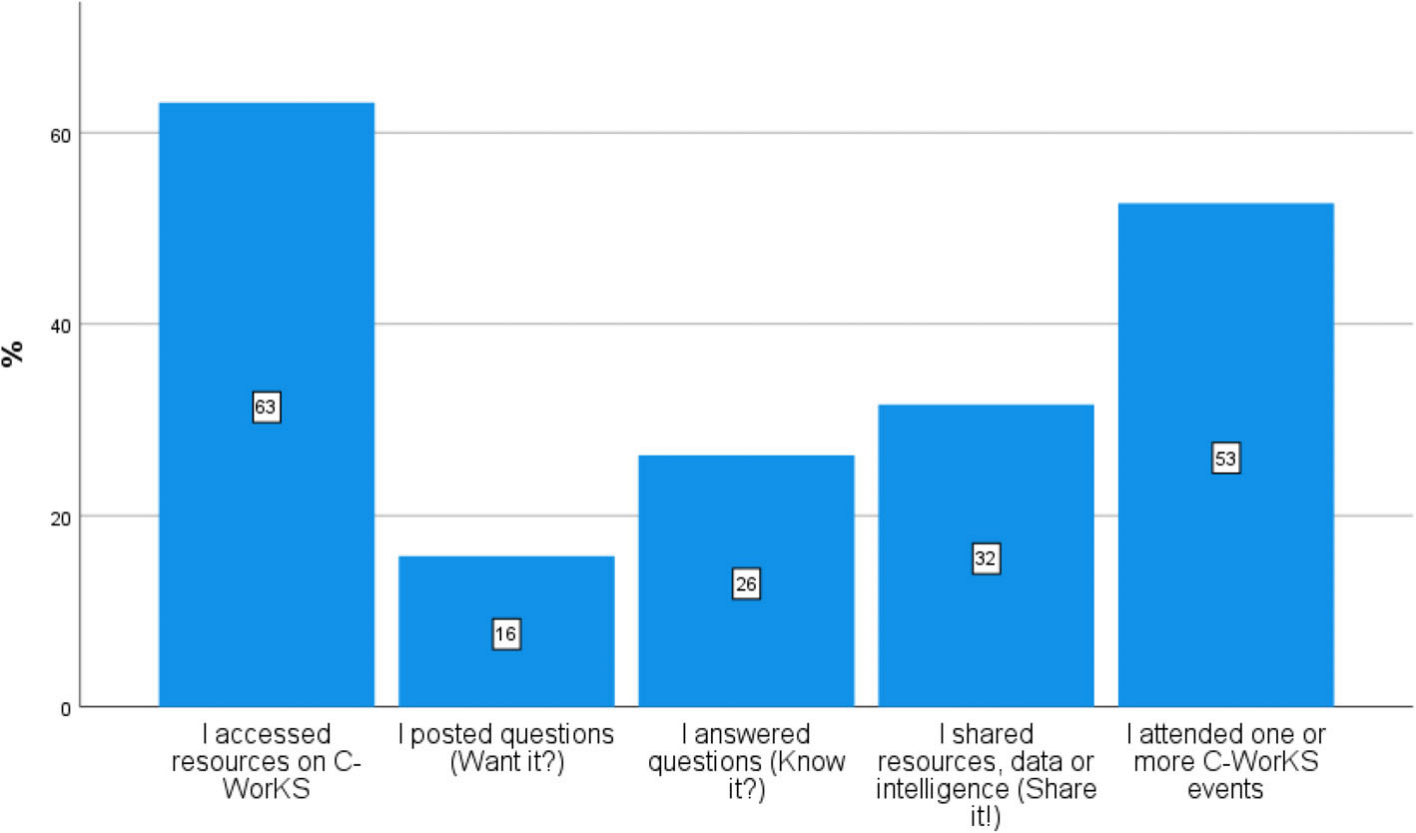
Level of engagement with C‐WorKS (*n* = 19). [Colour figure can be viewed at wileyonlinelibrary.com]

Of the 550 resources uploaded many appear to be uploaded by a small group of users and often involved members of RAG and MAPS. The social network analysis survey highlighted that most respondents only access resources on C‐WorKS (63%), with a small minority posting questions (16%), and a larger group answering questions (26%) or sharing resources (32%) (Figure 3).

This is supported by analytical data on individual page visits and contributions added by members to C‐WorKS, with between 23% and 31% of visitors contributing to the platform and community (Table 1).

The active support from RAG and MAP members in responding to questions posted on C‐WorKS, appears to have created a concentrated environment that hinders the ultimate goal of the platform: to provide a self‐sustaining community across organisational and hierarchical boundaries.

It seems like they're quite happy for us to put stuff on, but I sort of feel like there should be more of a two‐way thing, because we want people to share with us and with the networks what they've been doing and what they found. (stakeholder)

**TABLE 1:** Visits and contributions to C‐WorKS per year.

Year	Visits	Contributions^a^	% (contribution of visits)
2020–2021	2977	920	30.9%
2021–2022	3412	766	22.5%
2022–2023	2758	359	13.0%^b^

^a^ Contributions include adding resources, leaving comments, uploading posts to the forum.

^b^ Data for 22/23 is incomplete with data from the final quarter missing.

In addition, the current KHub platform limits the ability to collect more specific user data on C‐WorKS, particularly on platform access and use; thereby hampering the project team's ability to inform the future development of the platform and community.

There was a lot of time and effort that went into it and not knowing the impact that was happening. I think it was a bit of a blind side, because we were counting the number of resources and the people going on. But like I say, that doesn't necessarily translate into people reading using the resources, reducing the duplicate duplication of effort. So, we're very much counting things in the process book that didn't actually tell us what impact it was having. (stakeholder)

This lack of ability to collect data on the platform also caused delays in the research, as it was difficult to assess the level of user engagement when developing a sample pool for the interviews. For example, it was not possible to distinguish between members who only engaged with the wider PHINE community but not with C‐WorKS, which had to be checked manually in follow‐up conversations with interviewees.

### Opportunities

Over time, and in part in response to limitations of the online platform raised through real‐time user feedback, broader engagement activities for users outside the on‐line platform evolved (newsletters, resource kits on specific topics such as health inequalities curated by a librarian, online learning and training resources, social media promotion, and online webinars and workshops). These activities have created new opportunities for widening engagement. These additional services have proved to be popular among users, creating a community around the platform that is driving new users to the platform. In particular, the weekly email summaries were much appreciated by users, as a way of accessing new content and resources more quickly, without the need to login on C‐WorKS to explore content.

Some people are engaged with the workshops. But then often what happens with the workshop is people think “actually that's really interesting. I want to read more. I want to learn more” and then that then made people think. “Oh actually, I will register with the platform and look at the health inequality's impact assessment document now possibly before they go into workshop”. [..] definitely the workshops have catalysed interest in the platform. (stakeholder)

**FIGURE 4:**
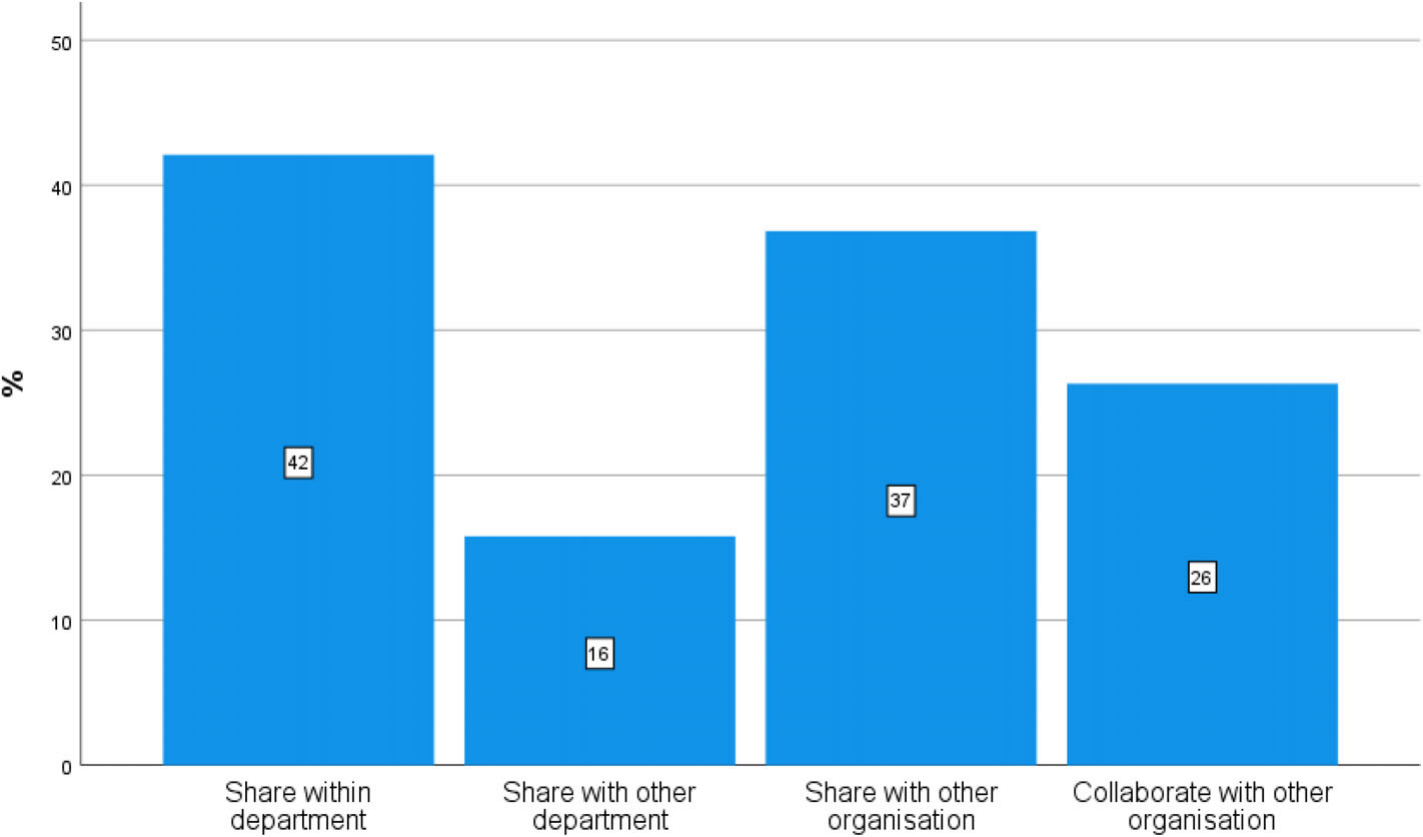
Sharing C‐WorKS evidence with colleagues (*n* = 19). [Colour figure can be viewed at wileyonlinelibrary.com]

The social network analysis survey suggests that C‐WorKS has a good reach with more than a third (37%) of respondents approaching colleagues outside their organisations about accessed resources, and over a quarter of respondents (26%) reported that these conversations let to further collaborations based on the evidence accessed through C‐WorKS (Figure 4).

At the same time as building a community around C‐WorKS, increasing engagement with C‐WorKS as a digital platform and its resources remains paramount to securing its future. This requires a different hosting platform, with greater accessibility, navigation and sharing options for users, and more specific monitoring functions for the project team that are currently not possible on KHub. Stakeholders have suggested the FutureNHS Collaboration Platform as an alternative; however, this platform might limit access for health professionals outside NHS organisations, such as local authorities and voluntary community sector organisations, as the registration process for these individuals to use the platform is more complex.

I had wanted C‐WorKS to have more people commenting and raising questions. There's an equivalent platform called NHSFutures, which is the NHS version almost of KHub, and I'm on a couple of groups there and there's loads of activity, loads of people posting questions, and asking, and people replying, which is really nice; to see people having that conversation. I don't think we we've managed to get enough of that in the C‐WorKS, and it might be to do with KHub itself not being a very good platform—I don't think people find it easy to navigate. (stakeholder)

Further opportunities exist around developed resources on new topics, such as health inequalities, to keep the platform attractive to existing and future users. Health inequalities is by far the most popular theme on C‐WorKS according to the social network analysis survey with 89% of respondents accessing evidence from this theme. This is also reflected in the large number of resources posted within the health inequalities category. The newly established Integrated Care Boards (ICBS) within NENC & Yorkshire might provide a wider regional structure for identifying and prioritising new topics on C‐WorKS, and also a useful partnership for C‐WorKS to draw resources from for delivering their various services and activities, tailored to this new structure.

For me as a commissioner across a system, particularly as we're moving into the Integrated Care System (ICS) where we are now making big decisions that are going to have implications for many years. We've got five‐year plans to be pulled together over the next 12 months; there is something about what could C‐WorKS do to support ICSs, to develop prioritization mechanisms that enable us to make objective decisions based on health improvement and health inequalities reduction? [..] some real hard hitting stuff that we can do around projections. [..] that would be really powerful. (user)

The social network analysis survey suggests that C‐WorKS should first and foremost connect people and solutions to their problems (42%), emphasising the important knowledge brokering role that C‐WorKS can continue to play and develop in the NE&Y.

### Threats

The lack of work‐loaded time for RAG and MAPS members and lack of funding for developing resources and events creates a threat for the future of C‐WorKS in case staff time is reduced, staff move on, and/or organisational support and goodwill for C‐WorKS is withdrawn. To ensure the sustainability of the platform and community, urgent funding is required to secure dedicated capacity for maintaining and further developing C‐WorKS.

The limited number of members actively engaging with C‐WorKS puts further pressure on the project team to maintain and develop the platforms and makes the business case for sustainability funding more challenging. Encouraging more active involvement from members through the wider community activities is therefore key for the sustainability of the platform, while ensuring that these activities drive more traffic to the platform.

The current KHub platform is limiting engagement between the wider community that engages with activities outside the platform and the online resources and tools available on the platform itself, hampering future development.

## DISCUSSION

### Key findings and transferable learning

Our analysis identified several strengths, weaknesses, opportunities, and threats in the development and implementation of an online knowledge sharing platform and community in the NE&Y, which supported joint learning, collation and sharing of knowledge and intelligence across the region about impacts of COVID‐19 (and responses to this) on non‐COVID related disease and death (Table 2).

**TABLE 2:** SWOT analysis of C‐WorKS.

*Strengths*: Rapid development in response to COVID‐19 pandemic (3 months) Active governance structure with strategic and operational groups Collaborative decision‐making with partners Working across organisational boundaries	*Opportunities*: Expanding the community of users and producers beyond the online platform Additional services & communications More accessible hosting platform New topics and wider support structures
*Weaknesses*: Build on goodwill of project team members and key users Current platform not user friendly Engagement from users is limited Lack of useful monitoring data	*Threats*: Lack of funding and work‐loaded time to sustain C‐WorKS Only active engagement with the platform from a core group of users Limited links between platform and wider community

Our SWOT analyses provides transferable learning for other knowledge sharing platforms and communities of practice. In this way we intend the findings to be generalisable so that they can inform others working in non‐COVID related fields, who are also trying to facilitate collaboration and information sharing across a diverse network.

A useful framework for identifying transferable learning is provided by a recently published independent review by NHS England into research, innovation and partnership working in relation to the pandemic response (BCN/ ACC, 2022). The review was jointly commissioned by the North East and North Cumbria Beneficial Changes Network (BCN) and the Accelerated Access Collaborative (AAC). The review aimed to understand the impact of the response to the pandemic in relation to health innovation, health research and partnership working across health and care in England, to learn lessons from this period and recommend how potentially beneficial changes can become day‐to‐day practice. The review identified six core findings as described in Table 3 below.

**TABLE 3:** Pillars of successful health research and partnership working (BCN/ ACC, 2022), as evidenced in C‐WorKS.

Core review findings	Description	C‐WorKS evidence
1. Clarity of purpose	A system‐wide shared understanding of the need for action mobilises partners quickly and breaks down barriers to collaboration	C‐WorKS addressed a clear system‐wide need for knowledge sharing about the impact of COVID‐19 on non‐COVID related disease and death
2. Leadership	Beneficial change is accelerated by leadership across organisational levels, and supports innovation and collaboration	C‐WorKS was rapidly developed and implemented through an active governance structure
3. Inclusion and personalisation	Addressing health inequalities requires greater inclusion and involvement of diverse perspectives, and the better personalisation of services to different populations	C‐WorKS's governance structure included a range of organisations and key stakeholders across the health and care sector in the North East and Yorkshire
4. Skills and capability	Change was enabled by those who had appropriate skills to solve problems, then adapt to new ways of working	Project team members brought a range of skills for the development and implementation of C‐WorKS; however, a lack of work‐loaded time and funded resources threaten the sustainability of C‐WorKS
5. Data and technology infrastructure	Critical enablers of rapid change include the safe and timely sharing of data, and appropriate and resilient technology infrastructure	The existing Khub platform supported rapid development and sharing of data; however, the limited user friendliness is hampering future development, and not effectively linking the community being built outside the platform to the online resources and tools
6. Evidence‐based decision‐making	For the impacts over time to be fully understood, there is a continuing need for robust evaluation evidence to understand what works, for whom and under what circumstances	Our evaluation contributed to the sixth core finding, by evaluating the implementation, development and impact of C‐WorKS

Our evaluation findings highlight that C‐WorKS addressed all six pillars of the review findings. What our evaluation adds to the six core findings identified by the BCN/ ACC review, is a focus on balancing leadership with inclusion and personalisation for the development and implementation of online knowledge sharing platforms. Striking a balance between facilitating a community and enabling self‐organisation to happen is an ongoing challenge for the development of C‐WorKS. Leadership is required to get responses to posted questions going, but at what point can this role be handed over to the community and how can this hand‐over be best facilitated and embedded into the processes and structures of C‐WorKS? And what is the potential risk to the platform and community if individual members don't get more involved in leadership roles? This requires culture change in health organisations to enable these roles and practical support with handover of responsibilities between community members over time.

### Relation to previous literature and recommendations

This dilemma deepens current understanding in the literature about community members remaining hesitant about their capacity as individuals to design and perform important knowledge exchange activities independently on knowledge platforms, as reported by (Thomson et al., 2019). Our study suggests that a change in leadership style might be required as the work develops (7th pillar), with an initial period of stronger top‐down leaderships to develop and implement C‐WorKS, complemented by more distributed forms of leadership (Kislov et al., 2023 (forthcoming)) that allow different members in the community across organisations to make decisions about content and process for generation and updating this content.

This shift in leadership style can contribute to the sustainability of online knowledge sharing platforms by continuing member involvement, which has been reported in the literature as a problem. For example, Kothari et al. (2015) found that community members were sometimes faced with resistance at organisational or governmental levels when implementing recommendations into practice. This reduced enthusiasm and motivation for members to stay involved. Enabling more distributing leadership for the platform across organisations secures buy‐in from senior management from the start for implementing recommendations and practice changes emerging from platform interactions between members.

Secondly, our findings confirm the need to utilise and link a range of online knowledge sharing techniques to engage with different audiences and sustain their involvement with online knowledge sharing platforms. Particularly, building on user access to the platform by creating a community of practice outside the platform for mobilising resources between health organisations.

For me it was great to see C‐WorKS branching out from just being this, what could even be called a sterile online platform to a community that interacts in a range of ways, as well as being a platform. We have a workshop. We have a newsletter, we have communications. We have even Twitter. It was great to see it almost grow legs in terms of a concept, and for me the way that the MAPS group have taken on the concept of community and how you relate to different people and how people want to interact in that space. (stakeholder)

These findings validate suggestions made by (Mairs et al., 2013) to overcome challenges related to access to and user knowledge of online technologies. Communities of practice developed through events and workshops provide an accessible social context for health professionals to access and understand resources available on the knowledge sharing platform and reduces information overload (Hall & Walton, 2004) when searching for health‐related information online. Other literature suggests to use of existing social media such as Twitter, Facebook and WhatsApp to support health practitioners in sharing knowledge and developing online communities (Keir et al., 2021). These readily available media have only been used in the promotion of C‐WorKS and could be used more actively to generate discussion among members to further enhance online knowledge sharing.

### Limitations of the study

As an explorative study with small sample sizes for the stakeholders and user interviews and limited responses to the online survey, the findings can only be indicative of the knowledge sharing between users and use of knowledge outside the platform. While a low response rate is not uncommon in online surveys and we took several steps to increase response rate, including personal approaches through network contact and offering vouchers as an incentive, we feel that the pressures on local authority and NHS staff due to COVID‐19 severely limited participants ability to engage in the research. Particularly, the low response rate to the survey limited the description of the network, allowing us to use only qualitive descriptors and no mathematical denominators. However, the survey findings provide important insights into the use of knowledge and intelligence between users outside of the C‐WorKS platform. Our study confirms and extend findings from the existing literature, which suggests a degree of validity of the results.

Our study was only able to focus on the short‐term outcomes of C‐WorKS (how evidence is shared through C‐WorKS), while for future development of C‐WorKS and similar platforms it would be beneficial to know what the long‐term impact of this evidence sharing is. For example, by following up users to assess how evidence use is sustained through the networks of relationships supported by C‐WorKS, and how this evidence use informed the design and implementation of public health interventions in responses to COVID‐19. Ultimately, the main aim of C‐WorKS was to reduce mortality and morbidity due to the non‐COVID consequences of COVID, which is a long‐term outcome that requires ongoing research.

## CONCLUSION

Collaboration supported by online knowledge sharing provides pragmatic and timely strategies for health professionals in the UK to apply research evidence to their practice. Our study provides practical insights in how to develop and implement a knowledge sharing platform, combined with a community of practice, addressing challenges identified in the literature around leadership and sustainability. We show, in addition to the six pillars of innovative health research partnerships, the importance of actively considering the need for evolving leadership styles during the life course of a knowledge sharing platforms and communities. With the increased importance of data and intelligence post‐pandemic, and health professionals lacking capacity to systematically search for and apply this data, the knowledge brokering role of these platforms and communities will only increase. Future research could focus on the long‐term effects of knowledge sharing on practice change and ultimately health outcomes, while comparing functionality and hosting mechanisms across platforms to assess how this impact on user engagement.

## FUNDING INFORMATION

This study/project is funded by the National Institute for Health and Care Research (NIHR) Applied Research Collaboration (ARC) North East and North Cumbria (NENC) (NIHR200173). The views expressed are those of the author(s) and not necessarily those of the NIHR or the Department of Health and Social Care.

## CONFLICT OF INTEREST STATEMENT

Helen Park and Sarah Sowden were members of RAG and have been involved in the development and implementation of C‐WorKS. In this role, they provided valuable insights for the protocol development and conduct of study while not inferencing with the independence of the research. As co‐applicants for the funding proposal and in the spirit of co‐production of the research, they are included as co‐authors on this paper, having contributed considerably to the writing of this paper.

## Supporting information


**Appendix 1.** INTERVIEW SCHEDULE—C‐WorKS stakeholders.
**Appendix 2.** INTERVIEW SCHEDULE—C‐WorKS users.
**Appendix 3.** Social Network Analysis (SNA) Questions.
