# Supplementary material for: Developing an online knowledge sharing platform and community of practice for health professionals: Experiences from C‐WorKS developed in North East England and Yorkshire during COVID‐19
Source: Health Info Libr J. 2024 Feb 1;42(2):177–89. doi: 10.1111/hir.12519 (PMC12723334; doi:10.1111/hir.12519)
Supplement: Supplementary file 1 — Appendix 1. INTERVIEW SCHEDULE—C‐WorKS stakeholders. Appendix 2. INTERVIEW SCHEDULE—C‐WorKS users. Appendix 3. Social Network Analysis (SNA) Questions. [file HIR-42-177-s001.docx]

## **Appendices**

### **Appendix 1. INTERVIEW SCHEDULE – C-WorKS stakeholders**

**Experience and context**

- Can you tell me a bit about your role within the development and ongoing roll out of C-WorKS?
- What experience do you have in developing knowledge and information sharing services?
- What types of evidence/ knowledge have you used in the development of C-WorKS? (Prompts: did you use research evidence, local intelligence, tacit knowledge and/ or practical wisdom? Who supplied this evidence and in what format?)
- How helpful where these different types of evidence/knowledge for informing your decision making about the C-WorKS platform?

**Membership of the working group**

- How would you describe the review and act groups approach to developing C-WorKS?
- When did you join the review and act group?
- How did you become a member of the review and act group? (Prompts: where you invited to join, or did you volunteer for the role? Who asked you/ provided you with information about the group and the C-WorKS platform?)
- How do you see both your role and the role of the working group in developing, implementing and evaluating the platform and its outcomes? (Prompts: What do you aim for C-WorKS to improve? what do you think you contribute to the group?)

**Development**

- Can you give an example of something you feel works effectively on the C-WorKS platform.
- Was there anything you felt was less effective or did not work as originally planned (prompt: did you rework the idea or was it removed from the platform?)
- In hindsight are there any elements of the C-WorKS platform you would have developed differently or made changes to?
- What change do you see C-WorKS taking as it continues to develop? (Prompt: what future ideas do you have for the development of C-WorKS?

**Any other questions?**

Do you have any other questions or comments that you would like raise?

### **Appendix 2. INTERVIEW SCHEDULE – C-WorKS users**

**Introduction**

- How would you describe C-WorKS?
- Have you used online knowledge sharing platforms before? If so, which ones and how would you compare these platforms to C-WorKS? (Prompt: what is unique about C-WorKS?)

**Joining**

- How did you hear about C-WorKS?
- How long have you been a member of C-WorKS group?
- Why did you choose to become a member of C-WorKS and what did you aim to achieve?

**Functionality**

- How did you find the joining process for C-WorKS group on K-hub? Were you already a k-hub member?
- How do you find the functionality of the C-WorKS group?
  - How do you find navigating the group and its pages?
  - How easy or difficult is it to find what you need?
- What aspects of C-WorKS do you primarily access?
  - Have you ever attended any of the workshops/ webinars hosted by C-WorKS?
    - Were they informative?
    - Interesting guest speakers?
    - Relevant to area?

**Outcomes**

- Did you need access to specific knowledge and intelligence resources related to the non-covid consequences of covid which you have not be able to access via c-works?
- How have you utilised the tools and resources provided via the group? (if not why?)
  - - Can you give an example of this?
    - How did they impact on your professional practice? Was there a change to your delivery for patients/the public?
- Have you expanded your professional network by making contact with new people or organisations using C-WorKS resources?
  - - Did this impact on the service you were able to provide to patients / users? If so, how? Can you give any examples?
- What do you think you contribute to the group? (Skills, knowledge, etc.)
- Have you posted any resources yourself on C-WorKS? If so, which ones?
- Have you responded to any queries raised by other platform users? If so, which ones and what did you contribute?

**Future**

- How you like to see C-WorKS develop in the future?
  - Features
  - Tools
  - Additional services
- What would encourage you to make more use of the platform?

**Any other questions?**

Do you have any other questions or comments that you would like raise?

### **Appendix 3. Social Network Analysis (SNA) Questions**

**Introduction**

In the following short survey, we ask you a few questions about yourself, how you view and use evidence, and what you think our priorities should be for C-WorKS. All information you give is treated in confidence. We will not identify people by name in the reports or publications we produce. More information about the evaluation project and how you can contact us with any questions can be found in the Participation Information Sheet by following this link (insert GoogleDocs link to PIS). By clicking on the next button, you consent to take part in the survey and for your data to be used in the research. As the survey is anonymous, you will not be able to withdraw your data. Thank you very much for your time!

1. **Please tell us where you work**

| Local Authority 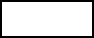  NHS 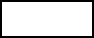  Voluntary sector organisation 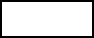  Public Health England 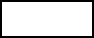  NIHR Clinical Research Network 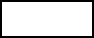  University 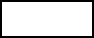  Other, please specify …………………………………………………………………………………. |
| --- |

***1b. Would you mind sharing the name of your organisation with us?*** …………………………………….

***1c. If applicable, which department in this organisation do you work in?***

……………………………………

1. **How long have you worked there?**

Less than 1 year
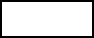
 1-5 years
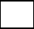
 6-10 years
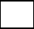
 11+ years
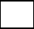


1. **How do you define evidence? Please tick as many boxes as relevant**

| Local monitoring data 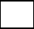  Local service evaluations 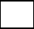  Joint needs assessment / future needs assessment 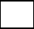  Public health surveillance data (e.g. from PHE) 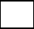  Practice guidelines (e.g. NICE) 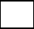  Systematic reviews (e.g. Cochrane) 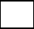  Published scientific papers 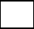  Case reports/ studies 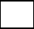  Other (please give details) ……………………………………………………………….. |
| --- |

1. **Where do you access evidence?**

| Public Health England 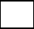  Government websites (e.g. DHSC) 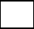  NICE 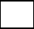  Cochrane 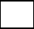  Professional bodies (e.g. Local Government Association) 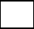  International organisations (World Health organisation) 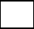  Online academic journals 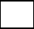  Universities (please specify which) 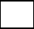  C-WorKS 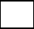  Experts in the area 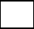  Other people (colleagues, friends) 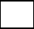  Other (please specify) 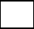 |
| --- |

1. **How do you use evidence in your work?**

| To plan services / joining up of services 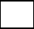  To inform commissioning decisions 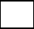  To understand what works to improve people’s health 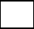  To help target public health interventions 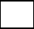  Other (please specify) ……………………………………………………………………… |
| --- |

1. **How has COVID19 changed the ways in which you access and use information?**
2. **From which themes have you accessed evidence on C-WorKS (Please tick as many boxes as relevant)?**

Health inequalities
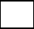


Inclusive economic growth
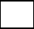


Recovery
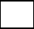


Health service disruption
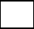


Population Health Management
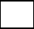


Mental health and well-being
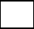


Wider health and care services
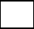


Wider determinants of health
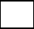


Health-seeking behaviours
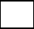


Health Literacy
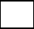


Excess Mortality
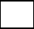


Beneficial Changes
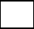


Staff and organisational issues
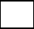


1. **How have you engaged with C-WorKS? (Please tick as many boxes as relevant)**

I posted questions (Want it?)
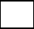


I answered questions (Know it?)
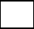


I shared resources, data or intelligence (Share it!)
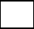


1. **Did you contact any individual inside your department to share or discuss the evidence you accessed on C-WorKS with?**

Yes
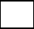


No
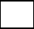


I don’t know
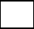


- 1. If so, what is the role / job title of the individual or the name of the team/department you have contacted?

………………………………………………………………………………………………………………………………..

1. **Did you contact any individual in another department in your organisation to share or discuss the evidence you accessed on C-WorKS with?**

Yes
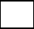


No
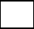


I don’t know
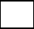


- 1. If so, what is the role / job title of the individual or the name of the team/ department you have contacted?

………………………………………………………………………………………………………………………………..

1. **Have you contacted any individual / group / organisation outside your organisation to share or discuss the evidence you accessed on C-WorKS?**

Yes
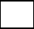


No
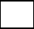


I don’t know
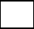


- 1. If so, what is the name / job title of the individual or the name of the group or organisation you have contacted?

………………………………………………………………………………………………………………………………..

1. **Do you collaborate with any organisations or groups based on the evidence you access through C-WorKS?**

Yes
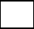


No
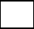


I don’t know
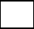


- 1. If so, what is the name of the organisation with whom you collaborate?

………………………………………………………………………………………………………………………………..

1. **Are there any organisations you think it would be useful to contact to share or discuss the evidence you accessed on C-WorKS?**

Yes
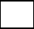


No
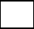


I don’t know
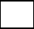


1. If so, please state which organisation? ………………………………………………………………………………………………………………………………..
2. **What are the three most important things for C-WorKS to achieve? Please mark three boxes 1,2,3 in order of preference, where 1 is the most important**

| Connect people and solutions to their problems 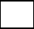  Increase understanding of what works to reduce the impact of COVID on  people’s health and wellbeing 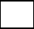  Develop shared resources on how to respond to COVID-19 effectively 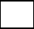  Build internal networks within organisations for sharing evidence 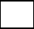  Build networks between local authorities, NHS, 3^rd^ sector and universities  Increase understanding of evidence needs in different organisations 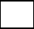  Embed evidence-informed practice and decision-making in organisations 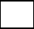  Improve readiness to collaborate during and after the pandemic between  individuals and organisations 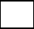  Facilitate changes in practice and policy (e.g. services delivery and  commissioning) 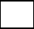  Other (please state) ………………………………………………………………………………. 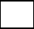 |
| --- |

1. **Do you have any other comments?**
2. **If you would like to receive the findings from this survey, please provide us with your email address and will we send you a copy of the research summary. Your email address will be stored separately from your survey answers to keep your data anonymous.**

………………………………………………………………………………..

**Thank you for completing this survey!**
